# Medical History from the Earliest Times

**Published:** 1892-10-22

**Authors:** E. T. Withington


### Medical History from the Earliest Times.

By E. T. Withington, M.A., M.B. Oxon.

### XXVIII.—Arabic Medicine. (1) The Translators.

In the year 632 there issued from the deserts of Arabia a people not entirely uncivilised, though classed by their more cultured neighbours as barbarians, armed with the tremendous forces of religious enthusiasm. Within a century they had annihilated one of the great existing empires, and inflicted a deadly blow upon the other; and after conquering Persia, and stripping Eastern Rome of her fairest provinces, had threatened the very existence of the younger nations of the West, till their career of conquest was checked at last by Frankish valour at the gates of Tours (732).

This display of physical vigour was followed by an intellectual activity hardly less wonderful. A Byzantine emperor was astonished to find that the right of collecting and purchasing Greek manuscripts was among the terms dictated by a victorious barbarian, and that an illustrated copy of Dioscorides was the most acceptable present he could offer to a friendly chieftain. The philosophers of Constantinople were amazed by the appearance of Moslem writers, whom they styled with reluctant admiration "learned savages," while the less cultured Christians of the West soon came to look upon the wisdom of the Saracens as something more than human. It was this people who now took from the hands of unworthy successors of Galen and Hippocrates the flickering torch of Greek medicine. They failed to restore its ancient splendour, but they at least prevented its extinction, and they handed it back after five centuries burning more brightly than before.

The cradle of Arabic medicine was the Nestorian school at Gondisapor, where the first distinguished Arab physician, Harets ben Kaladah, received his education. After practising with success at the Persian Court he returned to Arabia, and became medical adviser to the Prophet himself, who recommended him to his successor, the first Caliph, Abu Bekr. Harets was a Christian, a fact of great importance for the future of Arabic medicine, for even the strictest Moslem doctors confessed that the faithful might lawfully follow the example and precept of the Prophet in using the medical services of unbelievers. The first of the five centuries (750-1250) during which Arabic writers represented the highest form of civilised medicine, was devoted to translation. The translators were all Christians, most of them being connected with the school of Gondisapor, but we shall here confine our attention to the distinguished Syrian families of Bachtishua (Bocht-jesu) and Mesue, and the Christian Arab, Honain ben Isaac.

In the year 765 the Caliph al-Mansur, while engaged in founding the city of Bagdad, was seized with an attack of indigestion, and his friends urged him to send for George Bachtishua, Superintendent of the hospital at Gondisapor, and the most famous physician in Persia. George came, saw, and cured the patient, and the delighted Caliph retained him at his Court for five years, till, smitten with a mortal disease, he begged to be allowed to die at home. Al-Mansur then tried to convert him to Islam, promising him the joys of paradise, but George replied that he would rather go to his ancestors, whether in heaven or hell; so he was dismissed with rich rewards and a special escort, who brought him yet living to Gondisapor. His son also practised with success at Court, but is less important than his grandson, Gabriel, for more than twenty years physician to the great Harun al-Rashid and succeeding Caliphs, who, after many changes of fortune, could estimate in his old age that the total value of the fees and presents he had received exceeded £2,000,000. His son, Bachtishua ben Gabriel, was perhaps the most famous of the family, and Oriental historians have much to tell of his favour with the Caliph, Motawakkel (847-861), of the wonderful ebony chariot in which he drove through the streets of Bagdad, of his luxury and liberality, and of his terrible downfall. Once, while seated with the Caliph on his throne, the latter observed a hole in the physician's dress, and proceeded to enlarge it as far as the girdle. While thus employed, he asked, "How do you doctors know when a man is mad enough to be put under restraint?" "When he has torn our dresses as far as the girdle," was the reply. Then Motawakkel laughed till he fell on his back, and, on recovering, ordered the physician to be clad in a robe of honour.

For some time Bocht-jesu lived in a manner worthy of his name (Bocht-servant), but at last, led astray by Moslem associates, he fell into sin, and married two of his slave girls on the same day. From that time nothing went well with him. He spent his nights in orgies of wickedness, and his mornings in prayer, repentance, and reading the Gospel; he lost the favour of the Caliph, was deprived of his wealth, and banished to South Arabia, where he wandered in penury and loneliness till he died.

The family of Mesue was closely connected with that of Bachtishua, its founder, an apothecary and dispenser, at Gondisapor, having been a protégé of George, who, noticing that he admired a female slave belonging to David ben Serapion (another Gondisapor physician and translator), bought her for £20, or £2 10s., according to another authority, and gave her to Mesue as his wife. He was an illiterate man, but having, after 30 years' experience of dispensing, gained an intimate acquaintance with drugs, he aspired to higher things, quarrelled with the Bachtishuas, went off to Bagdad, and set up as a physician. He was so fortunate as to cure the Grand Vizier's favourite of an ophthalmia, and the Caliph al-Rashid being soon afterwards attacked by the same complaint, the Vizier recommended Mesue. "*You* a physician," said Gabriel, surveying the former apothecary with contempt; but he cured the Caliph in two days, and was rewarded with a pension of £1 a week. Soon afterwards Harun's sister was taken seriously ill, the friendly Vizier again recommended the man who had been thirty years at Gondisapor, and Mesue and Gabriel once more met in consultation. The former declared the patient would die. "He lies," said Gabriel, and the prophet of evil was cast into prison to await the event, but on the death of the princess, Mesue was released and raised to an equality with Bachtishua, and the two physicians appear to have become more harmonious, for Gabriel's daughter married Michael, son of Mesue.

But Meuse's most famous son was John, known to the Christians as Mesue the Elder, who was appointed by that great patron of science, the Caliph al-Mamun, President of a College of Translators, and who himself founded a sort of academy for scientific disputations. He was probably more feared than loved by his subordinates, for his manners were abrupt and his temperament cynical. Once, when seriously ill, the Nestorian clergy came, as was their wont, to pray for him. "What are these rascals doing here?" asked Mesue. "We are praying that God will restore you to health." "A few pills," was the answer, "will do more good than all your prayers, though they go on till the Resurrection"; and he drove them out.

John was succeeded in the office of translator-in-chief by one of his pupils whom he had treated very badly; but we must resist the temptation to tell the story of Honain ben Isaac, and confine ourselves to a sketch of his work. The Bachtishuas and Mesues were Syrians, and their knowledge both of Greek and Arabic was probably imperfect, but Honain was a native Arab, and had spent two years in Greece learning the language and collecting manuscripts. His translations and commentaries were such as had never before been heard in Bagdad. Even the aged Gabriel attended his lectures, and professed himself edified, and he never received less than its weight in gold for any of his manuscripts. Honain translated most of the works of Hippocrates and Galen, the great compilations of Oribasius and Paulus, the "Timæus" of Plato, many works of Aristotle, the geometry of Euclid, and last, but not least, the Septuagint, from Greek into Arabic. He also wrote original works, one of which, the "Introduction to Medicine," was much read in the mediæval universities. He may not unfairly be classed among the greatest men of the ninth century, and he died "chief of the physicians of Bagdad" in December, 873.

